# Perceived barriers to the provision of preventive care among dentists of Udaipur, India

**DOI:** 10.4317/jced.51770

**Published:** 2015-02-01

**Authors:** Ramesh Nagarajappa, Sudhanshu Sanadhya, Mehak Batra, Hemasha Daryani, Gayathri Ramesh, Pankaj Aapaliya

**Affiliations:** 1 Professor and Head, Department of Public Health Dentisty, Rama Dental College and Hospital, A-1/8, Lakhanpur, Kanpur – 208024, Uttar Pradesh; 2 Assistant Professor, Department of Public Health Dentisty, Government Dental College, Jaipur, Rajasthan; 3 Senior Lecturer, Department of Public Health Dentistry, Pacific Dental College and Hospital, Airport Road, Debari, Udaipur – 313024, Rajasthan; 4 Senior Lecturer, Department of Public Health Dentistry, Hitkarni Dental College, Jabalpur, Madhya Pradesh; 5 Associate Professor, Department of Oral and Maxillofacial Pathology, Rama Dental College and Hospital, A-1/8, Lakhanpur, Kanpur – 208024, Uttar Pradesh; 6 Senior Lecturer, Department of Public Health Dentistry, Vaidik Dental College and Research Centre, Nani, Daman

## Abstract

Aim: To investigate the practice-, patient- and dentist related barriers to the provision of preventive dental care as perceived by dentists of Udaipur city, Rajasthan, India. Settings and Design:- A cross sectional descriptive survey was conducted among 120 dentists of Udaipur city, Rajasthan. Material and Methods: Mean Content Validity Ratio (CVR) was calculated as 0.87 based on the opinions expressed by a panel of total six academicians. Cronbach’s coefficient was found to be 0.88, which showed a high internal reliability of the questionnaire. The questionnaire consisted of demographic questions and 12 specific research questions. Statistical analysis used:- Student’s t-test and ANOVA test were applied for the statistical evaluation of means. Level of significance was set at 0.05. Results: The barriers correlated strongly with each other (0.60 to 0.85). A significant gender difference was observed in mean sums of scores of practice and patient related barriers. Practice, dentist and patient related barriers for very much hindrance were reported by 8 to 13%, 5 to19% and 0 to 29% of the dentists respectively. A significant difference was observed among mean of sum scores of practice and patient related barriers with age and experience. Qualification was significantly related to practice related barriers. Conclusions: Perception of dentists showed that patient related barriers were found to be the foremost to the provision of preventive care. Also, dentist’s attitude towards health promotion and disease prevention needs a radical transformation.

** Key words:**Dental care, dentists, patients, perception.

## Introduction

Health care makes up an extensive sector of service industry in developing countries and employ many people (1). The restoration/extraction ratio in India is very low as compared to developed countries (2). Therefore, more emphasis is being placed upon developing an evidence based approach to clinical care and treatment (3).

Despite the fact that the majority of oral diseases are preventable, dental services in India currently focuses primarily on the conservative management of existing diseases. High cost, dental fear or anxiety and the physical barriers are the major obstacles for community dwelling adults in obtaining regular dental services (4).

Comprehensive knowledge of caries process has altered the understanding about the caries management from traditional drilling and filling to non operative and preventive approach with active control of caries. Concurrent improvements in restorative methods and materials have also made it possible to preserve tooth structure (5).

It is essential to understand the reasons why general dental practitioners have or have not adopted preventive practices (6). Barriers of various types may hinder dentists from applying preventive measures. These barriers can be summarized as practice-, dentist- and patient related barriers (5). Among practice related barriers, dental insurance and psychology of the patient are considered by dentists as barriers for providing dental care. The market of dental services can be modeled conceptually as an interaction between supply and demand. In private sector, effective demand results from perceived need for care which are influenced by factors such as income and third party payments. Person with dental insurance is more likely to visit dentist than a non insured person (7). People in India are still arrested in traditional rituals and superstitions which changed their psychology for seeking dental treatment.

Dentist: population ratio is 1:2.5 lakhs in rural areas which is lagging behind the required numbers (8). There is paucity of dentists in rural areas; people still rely on quack for dental procedures. Secondly they still believe in myths of extraction rather than on preventive measures. These two barriers are considered to be major patient related barriers. In dentist related barrier, dentist may find preventive practice is not being prestigious or profitable for them or they may have negative concept of prevention (5).

Dental health professionals often experience difficulties when they try to help their patients acquire and maintain actions which are conducive to preserving their dental health. Dentists practice and patients each have their own set of characteristics that may impact on the preventive process (9).

The purpose of this study was to investigate practice-, patient- and dentist related barriers to the provision of preventive dental care as perceived by dentists of Udaipur city, Rajasthan, India.

## Material and Methods

-Study design and study population:- A cross sectional descriptive survey was conducted among all the 120 practicing dentists at Udaipur, Rajasthan.

-Exclusion criteria:- Non practicing dentists.

-Ethical approval and official permission:- Before the commencement of the study, ethical approval was obtained from the Institutional Ethical Committee and official permission was obtained from the authorities of the dental institutions. Written informed consent was obtained from those who fulfilled the eligibility criteria. 

-Pretesting survey:- Assessment of content validity was done which reflects a judgment whether the instrument included all the relevant or important domains or not. Mean Content Validity Ratio (CVR) was calculated as 0.87 based on the opinions expressed by a panel of total six academicians. When face validity was assessed, it was observed that 92% of the participants found the questionnaire to be easy.

Prior to finalizing the questionnaire, previously validated questionnaire was pilot tested among a convenience sample of 15 dentists. Upon completion of the pilot response format, each subject was interviewed to gain feedback on the overall acceptability of the questionnaire in terms of length, language, clarity, and on the feasibility of dentists completing and returning it. Cronbach’s coefficient was found to be 0.88, which showed a high internal reliability of the questionnaire. Based on this analysis, all necessary changes were introduced before the main study.

-Proforma details: -The proforma consisted of a self-administered and structured questionnaire including:

1. Demographic questions including name, age, sex and professional background (qualification -Bachelor of Dental Surgery (BDS) or Master of Dental Surgery (MDS)) and work experience.

2. Specific research questions: There were 12 close ended questions which were divided into 3 categories: Practice Related barriers (4 questions), Dentist Related barriers (4 questions) and Patient Related barriers (4 questions). The participant’s responses for each question were ranked according to 5 points Likert scale: very much, much, little, very little and not at all.

-Methodology: -On the pre-decided days, investigator visited the dentists at private dental clinics and two dental colleges. The purpose of the study was informed and explained to them and were given the questionnaires. They were requested to fill in the written consent form if they were willing to participate in the survey and return the response format within eight days. Response involved choosing the most appropriate response from each of the 5 alternatives. Confidentiality and anonymity of the respondents were assured.

-Statistical analysis: -Every item of the questionnaire was coded from 1-5 (very much to not at all) and the data was analyzed using the Statistical Package for Social Sciences version 11.5 software. The sum of these scores for each barrier, with a theoretical range from 0 to 16, served as an indicator of the dentist’s perceived strength of practice-, dentist- and patient-related barriers to the provision of preventive measures. Based on the distributions of the sum of the scores, three categories were defined for the perceived strength of each barrier: weak (0 to 7), moderate (8 to 13) and strong (14 to 16).

Cronbach’s alpha was calculated to assess the relationship between individual items in each of the three barriers, and the correlation between the barriers was assessed by Pearson’s correlation coefficients. The Student’s t-test and ANOVA test were applied for the statistical evaluation of means. Level of significance was set at 0.05.

## Results

The reliability coefficients for the relationship between the items in each of the three barriers were as follows: for the practice related barrier, 0.88; for the dentist related barrier, 0.81; and for the patient related barrier, 0.81. The barriers correlated strongly with each other; (correlation coefficients ranged from 0.60 to 0.85).

 Table 1 shows the distribution of study subjects by age, gender, qualification and experience. Of the total 120 dentists, 84 (70%) were males and 36 (30%) were females. Majority of them were in the age range of 30-35 years (n=56; 46.7%), MDS (n=67; 55.8%) and with less than 5 years of experience (n=72; 60%).

**Table 1:**
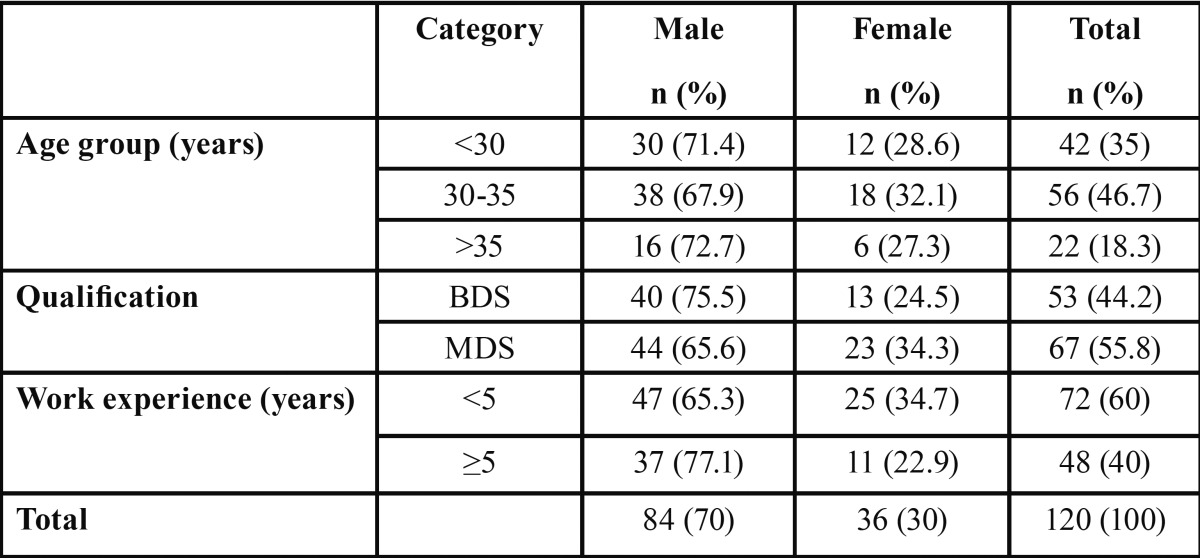
Distribution of study subjects by age, gender, qualification and experience.

The mean of score sums of practice, dentist and patient related barriers were 6.3 ± 3.3, 7.6 ± 2.2 and 8.0 ± 4.1 respectively. Practice, dentist and patient related barrier was opted as strong barrier by 11.7%, 10% and 22.5% of the dentists respectively. A significant gender difference was observed in mean of score sums of practice and patient related barriers ( Table 2).

**Table 2:**
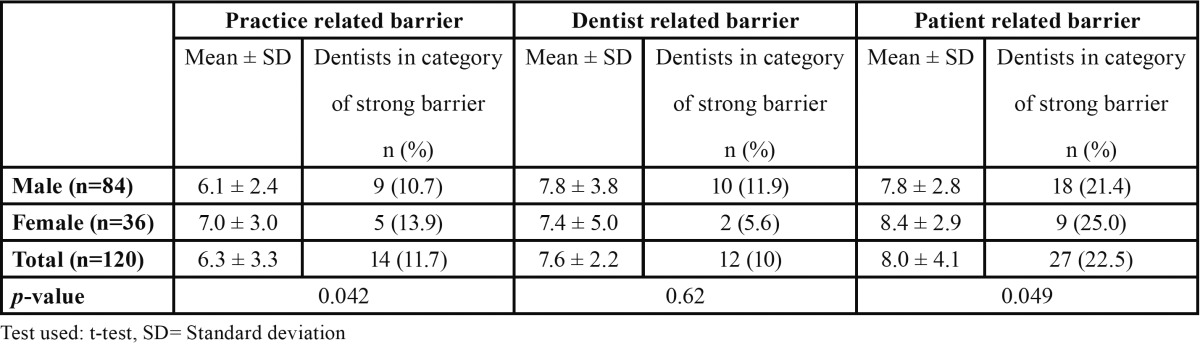
Mean of score sums of practice, dentist and patient related barriers according to gender.

 Table 3 reveals that mean of sum scores of practice related barrier was significantly lowest among >35 years age group (4.1 ± 1.3). Mean of sum scores of practice related barrier significantly increased with qualification and experience. Dentist related barriers did not show any significant differences. Mean of score sums of patient related barriers increased significantly with increasing age and years of experience and decreased with increasing qualification.

**Table 3:**
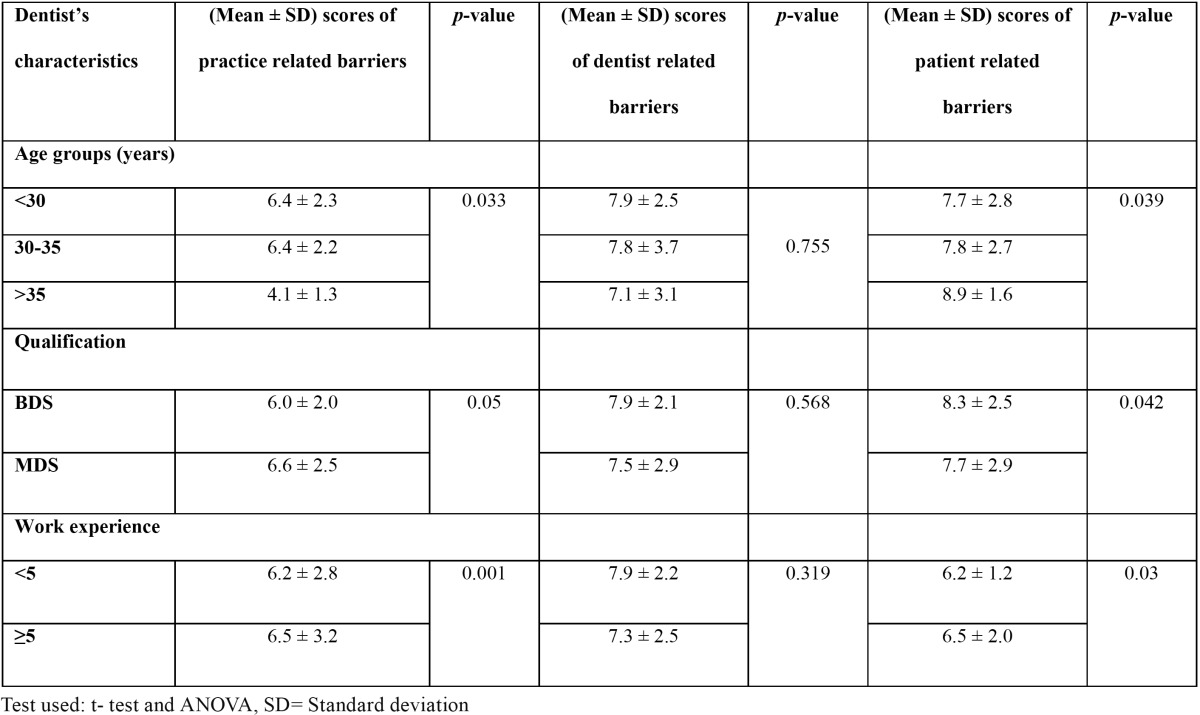
Comparative assessment of mean of score sums of the practice-related, dentist related and patient related barriers to the use of preventive measures according to the dentist characteristics.

**Figure 1:**
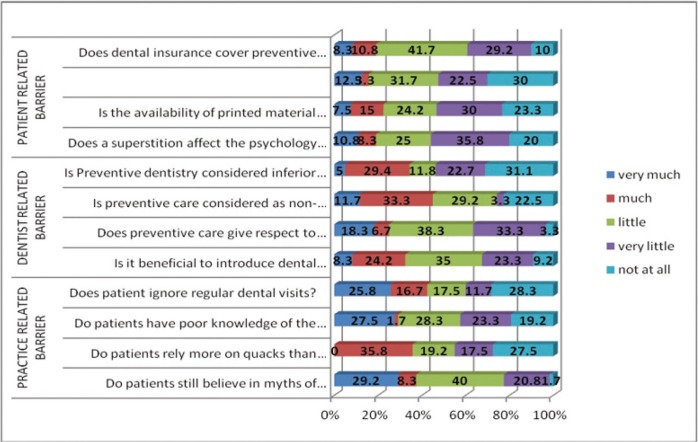
The percentage of responses showing dentists perceptions about practice, dentists and patient related barriers.

Figure 1 shows percentage of the responses to the 12 statements regarding barriers to the provision of preventive dental care. Among all, 8 to 13% and 3 to 15% of the respondents rated the practice related barriers as very much and much obstructive to preventive measures respectively. Dentist and patient related barriers accounted for very much hindrance by 5 to 19% and 0 to 29% of the dentists respectively.

About 10 to 30% of the dentists stated that practice related barriers do not inhibit them from carrying out preventive measures at all. Very much hindrance was reported by 29.2% of the dentists when they were asked about patient’s belief in myths of extraction.

## Discussion

Oral health promotion and prevention is critical to reducing disease burden and increasing quality of life. Preventive dental interventions, including early and routine preventive care, fluoridation, and sealants are cost effective in reducing disease burden and associated expenditures (10). Failure to provide access to preventive dental care almost always results in quick fixes that are short lived and high priced, especially among low-income children and their families who are without the resources necessary to access dental services (11). Prevention is the mainstay to avoid oral diseases and to have a positive oral health. This aspect of dentistry is the responsibility of professionals, individuals and society at large. Dentists are in a key position to help their patients to reduce the burden of oral disease and attain positive oral health behaviour.

The Federation Dentaire Internationale (FDI) suggested three separate categories of barriers to dental care. The first of these related specifically to individual and included: lack of perceived need, anxiety and fear, financial considerations and lack of access. The second category related to the dental profession. They included, inappropriate manpower resources, uneven geographical distribution, training inappropriate to changing needs and demands and insufficient sensitivity to patient’s attitudes and needs. The third and final category of barriers related to society, insufficient public support of attitudes conducive to health, inadequate oral health care facilities, inadequate oral health manpower, planning and insufficient support research. Apart from this, three types of barriers are identified pertaining to preventive dental care. These are: Practice Related Barrier, Dentist Related Barrier and Patient Related Barrier.

There is no reliable data available regarding major barrier to preventive dental care in India. Understanding the obstacles to change in dental practice is critical for the development of implementation strategies to assist the dental profession to respond to changing demands and circumstances. So this study was conducted on the dental professionals of Udaipur city to explore barriers pertaining to preventive care. Valid comparisons couldn’t be done with studies due to lack of literature, use of different types of questionnaires and studies done under different socio-economic status and cultural back grounds. However, wherever feasible comparisons have been done.

This cross-sectional questionnaire study has uncovered an interesting range of issues in relation to barriers hindering this area of preventive care within dental practices in Udaipur city. The high response rate (100%) of the respondents to this study guarantees that the subjects represent the target population dentists in Udaipur. A questionnaire survey was a suitable, economical and practical way for this type of data collection, although the tendency of giving socially more acceptable answers still remains. Questions were close ended, and statements are measured by means of a 5-point scale in order to improve accuracy of the analysis.

Most of the responses were in the category of “little or very little” for practice related barriers, whereas for patient related barriers it was “very much”. The dentist related barriers had maximum responses in the category of “much”. The most striking and pervasive issue emerging from the focus groups was the fatalistic and narrow concept in which prevention was viewed. The vast majority of dentists expressed frustrated and negative views on preventive dental care in general. Female dentists had a better approach towards preventive dental care compared to man counterparts as they had lesser dentist related barriers whereas the practice related barriers were very high. Lesser barriers were also encountered by female dentists in Mongolia, Iran and England. This may be due to less inclination of female dentists to economic aspects of practice. Female dentists tend to have greater interest in prevention as compared to their male counterparts and this may affect the dental practice and the employment of preventive measures (12).

As expected, the young dentists (fewer years of work experience) tended to disagree with their older counterparts about the different barriers interfering with their delivery of preventive care and similar reports were seen in other studies also (3,5,6).

The younger dentists (<35 years) have less patient and dentist related barriers but similar practice related barriers compared with more experienced dentists. The BDS graduates irrespective of practice have lesser practice related barriers but, more barriers related to patient and dentist for the provision of preventive dental care than the MDS graduates.

The dentists agreed that absence of dental auxillary is a barrier to deliver preventive dentistry. However, maximum response was in the category of “little” (35%). But in India, since there are very less professionally trained dental auxiliaries (dental technicians and dental hygienists); dentists need to put greater efforts into involving and cooperating with their auxiliary staff. For this purpose, the basic and continuing dental education should increase the number of courses in preventive dentistry and design special programmes to provide the knowledge and skills to practice a team work approach to prevention activites in the local circumstances (13).

Treasure *et al.* (14) found that, variations in (adult) disease are caused by more than social class structure, but certain behaviours are associated with social class. The highest numbers of young, exempt patient receiving preventive care may not reflect the social status of these individuals by accounting the reluctance of patients for payment. In our study we found a higher patient related barrier where in the patients believe in myths of extraction, rely more on quacks and home remedies, have poor knowledge of prevention of oral diseases and are highly irregular for schedules. India is a country with 75% of population in villages with higher illiteracy rate and lower accessibility to oral health care. This calls for understanding of psychological aspects of dentist-patient communication and relationships, active involvement of patients in assessment and evaluation, and giving realistic advice to patients. Dental attendance patterns are discouraging among patients in India due to time urgency or psycho-social factors such as dental anxiety, lifestyle and lack of accessibility to oral health professional to strengthen the alliance by formulating the treatment plans with which the dentist able to comply.

In this study, more than half the dentists claimed that insufficient remuneration deterred them from providing prevention. McCann *et al.* (15) found that 40% of respondent dentist were deterred from providing smoking cessation advice because there was no remuneration as shown by other studies also (16,17).

Lack of reimbursement system in India for dental care is the major hindrance for practice of preventive dentistry. So Government of India should suggest some directions to insurance companies regarding reimbursement system in preventive care. In Australia and USA, the likelihood of receiving preventive dental care is high among insured persons than in non-insured persons. It also confirmed a higher frequency of visits among insured patients and they had lower percentage of extractions and dentures (18).

From the present study we can recommend allocation of money and time for the promotion of preventive aspects and CDE programmes to be conducted for dentists about common risk factor approach. Emphasis should be given towards five principles of Ottawa Charter (19) viz; creating supportive environments, building healthy public policy, strengthening community action, develop personal skills, reorienting health services. Moreover, further studies with larger sample size, with different socio-cultural backgrounds are proposed.

In conclusion, this study has found that the main barriers to the provision of preventive care relate to patients attitudes, finances and treatment oriented culture among dentists. Dentists feel inadequately reimbursed for providing such care, and secondarily because they make assumptions about the type of treatment that their patients are willing to pay for. Many dentists appear unwilling to adopt new strategies such as the common risk factor approach, preferring instead to continue with basic oral health education irrespective of its impact on patient’s general health. It appears that many dentists need to be educated and convinced of the benefits of health promotion before they have the necessary information to provide appropriate advice to their patients. Changing circumstances require from the professional dental community in India, a change in its curative-oriented approach based on the earlier specialist-based dental care system towards a public health- and prevention- oriented one.
